# Evaluating the Involvement of People With Cancer and Informal Caregivers in the Development Process of a New Set of Quality of Life Questionnaires

**DOI:** 10.1111/hex.70267

**Published:** 2025-04-29

**Authors:** Merel Engelaar, Femke van Schelven, Nanne Bos, Marion L'Hote, Clémentine Rialland, Norbert Couespel, Carina Dantas, Caitriona Higgins, Tapani Kalmi, Inke Minnée‐van Braak, Laura Pinnavaia, Giovanni Apolone, Cinzia Brunelli, Augusto Caraceni, Montse Ferrer, Mogens Groenvold, Stein Kaasa, Gennaro Ciliberto, Claudio Lombardo, Ricardo Pietrobon, Gabriella Pravettoni, Aude Sirven, Hugo Vachon, Galina Velikova, Jany Rademakers

**Affiliations:** ^1^ Netherlands Institute for Health Services Research (Nivel) Utrecht the Netherlands; ^2^ European Cancer Organisation Brussels Belgium; ^3^ SHINE 2Europe Lda Coimbra Portugal; ^4^ Beaumont RCSI Cancer Centre Dublin Ireland; ^5^ Independent co‐researcher Turku Finland; ^6^ Independent co‐researcher The Hague the Netherlands; ^7^ Department of Languages, Literatures, Cultures and Mediations University of Milan Milan Italy; ^8^ Scientific Directorate Fondazione IRCCS Istituto Nazionale dei Tumori Milan Italy; ^9^ Palliative Care, Pain Therapy and Rehabilitation Unit Fondazione IRCCS Istituto Nazionale dei Tumori Milan Italy; ^10^ Department of Clinical Sciences and Community Health, Department of Excellence 2023‐2027 University of Milan Milan Italy; ^11^ Hospital del Mar Research Institute Barcelona Spain; ^12^ Bispebjerg/Frederiksberg Hospital and University of Copenhagen Copenhagen Denmark; ^13^ Oslo Universitetssykehus HF Oslo Norway; ^14^ IRCCS National Cancer Institute Regina Elena Rome Italy; ^15^ Organisation of European Cancer Institutes Brussels Belgium; ^16^ SporeData OÜ Tallinn Estonia; ^17^ Applied Research Division for Cognitive and Psychological Science European Institute of Oncology IRCCS Milan Italy; ^18^ Department of Oncology and Hematology‐Oncology University of Milan Milan Italy; ^19^ Unicancer Paris France; ^20^ Quality of Life Department European Organisation for Research and Treatment of Cancer Brussels Belgium; ^21^ Leeds Institute of Medical Research at St James's University of Leeds Leeds UK; ^22^ Leeds Cancer Centre St James's University Hospital Leeds UK; ^23^ Department of Family Medicine, Care and Public Health Research Institute (CAPHRI) Maastricht University Maastricht the Netherlands

**Keywords:** co‐researchers, Europe, oncology, patient and public involvement, patient participation

## Abstract

**Introduction:**

There is a general need for sharing practical examples of Patient and Public Involvement (PPI) within the research field to learn from and inspire. The aim of this article is to describe our process evaluation of PPI within the development process of the EUonQoL‐Kit, a new set of quality of life questionnaires aimed at people with (past experience of) cancer.

**Methods:**

Five co‐researchers (people with cancer and informal caregivers) were recruited and received training and support from a dedicated team of researchers. Involvement in the development process of the EUonQoL‐Kit consisted of four major events: two workshops, a consensus meeting and a stakeholder forum. We have collected event documents, that is, meeting agendas, presentation slides, minutes of the events and minutes of meetings with co‐researchers before and after the events, and qualitatively analysed these using the Cube Framework.

**Results:**

Our process evaluation showed that, over time, discussions evolved from focusing on the technical aspects of the EUonQoL‐Kit to co‐researchers' experiences as input for the questionnaires. Researchers' inexperience with PPI prompted the organisation of a training workshop. After this, researchers prepared the co‐researchers better for the meetings and engaged them more actively by asking specific questions. All these developments contributed to a more active participation of co‐researchers.

**Conclusion:**

PPI in the development process of the EUonQoL‐Kit was a learning process. Factors that helped include allocating time and resources, actively creating space for co‐researchers' input, providing support by researchers specifically responsible for the PPI activities and realising the importance of informal contact. Future PPI efforts should incorporate these principles from the start to facilitate successful collaboration between researchers and co‐researchers.

**Patient or Public Contribution:**

People with cancer and informal caregivers played a significant role in this study. They were involved as co‐researchers in all stages of the development process of the EUonQoL‐Kit. In addition, they were involved in the qualitative analysis of the data presented in this article, the writing of the project report and the writing of this article as co‐authors.

## 1 Introduction

It is becoming increasingly common to integrate patient and public perspectives in a wide range of research areas, including quality of life research and oncological research. In the field of quality of life research, patients have often been involved in the initial steps of the development process, for instance, as participants in interviews and focus groups. However, active collaboration with patients and the public has not yet been widely adopted [1, 2]. The development of patient‐reported outcome measures (PROMs) offers the potential for more collaborative forms of research, with patients or other people with relevant experience participating as active members of the research team, working together in all aspects of the development of a PROM to enhance its quality, relevance and acceptability [1]. This collaboration with patients and the public is also called Patient and Public Involvement (PPI) [3].

The literature provides many arguments for collaborating with patients and the public in health research [2, 3, 4, 5, 6]. According to moral arguments, having a say in decision‐making that will eventually concern them is everyone's fundamental right [4, 5]. Methodological arguments focus on improvements in the quality and relevance of research. PPI contributes to improved research questions and methodologies that better match the needs and wishes of patients and the public [6, 7]. Educational arguments suggest that PPI is an enriching experience for patients, public members and researchers, as they obtain new skills and knowledge through their collaboration [6, 8]. In general, PPI brings science and society closer together.

Achieving successful PPI that lives up to these expectations, however, is a struggle for many. This is the result of its situational and dynamic nature [9, 10]. PPI processes strongly depend on those involved and the context they take place in, which means that they are created in continuous interaction between all that are involved [5, 10]. Additionally, it is often not clear in advance how PPI processes will develop, which can cause feelings of discomfort among researchers who are generally used to setting protocols and plannings [11]. Some guidelines and best practices for PPI are available, including developing a good working environment, establishing good collaboration and taking into account individual and team differences [8]. It has also been recommended that sufficient effort, time and resources be devoted to conduct meaningful PPI and continuously discuss roles and responsibilities [12]. Additionally, it is advised to balance different forms of involvement and to utilise visual methods in PPI activities to facilitate the involvement and free thinking of patients and the public [13].

Although there is increasing attention to both the conduct and reporting of PPI activities, there is still much to be gained regarding knowledge and experience within certain research areas. A recent survey showed that only about half of cancer researchers had any relative experience with doing PPI, and a need for practical examples was articulated [14]. Furthermore, there is little evidence of how to implement PPI in large‐scale, multinational projects that operate in multiple linguistic and regulatory contexts [15]. A recent systematic review into approaches to PPI across Europe only identified two studies where PPI was focused at a pan‐European level, of which only one had cancer as the focus of the topic [16]. To further improve PPI in research, sharing practical examples can support and inspire the research field on how to organise collaboration with patients and the public and how to deal with challenges that are encountered [17, 18].

Therefore, the aim of this article is to describe the results of our process evaluation of PPI within the development process of a new PROM, consisting of quality of life questionnaires aimed at people with (past experience of) cancer, taking place in the European research project called ‘Quality of Life in Oncology: measuring what matters for cancer patients and survivors in Europe’ (EUonQoL). Additionally, we aim to articulate lessons learned for future research efforts beyond this study project.

## 2 Methods

### 2.1 The EUonQoL Project

The EUonQoL project aims to review existing quality of life scales to develop new metrics by harnessing the strengths and overcoming the limitations of previous tools. The EUonQoL‐Kit, a new PROM consisting of quality of life questionnaires designed for people with (past experience of) cancer in Europe, will be the product of this effort. It will form a new digital system for self‐assessing the quality of one's life, available in several European languages, developed from the patient's perspective and appropriate for assessing the whole cancer care continuum [19]. Input for the EUonQoL‐Kit consists of systematic reviews of existing quality of life frameworks and measurement tools, as well as a Delphi study and interviews to establish patients' priorities and preferences for quality of life domains. The EUonQoL‐Kit will be validated in a pilot study (Clinical Trials ID NCT05947903). Further description of the project and the participating organisations can be found on the EUonQoL website (http://www.euonqol.eu/).

### 2.2 PPI

The EUonQoL project is based on PPI research principles by involving individuals with an active or previous experience of cancer and informal caregivers as ‘co‐researchers’. In this project, the term ‘co‐researchers’ is used for those people who have experienced cancer, either as a patient or an informal caregiver, and who now collaborate with the researchers. Recruitment of co‐researchers took place via a call for action that circulated on social media (Facebook, X and LinkedIn) and through the newsletter of the Organisation of European Cancer Institutes. Potential co‐researchers who expressed their interest first received additional information via email and were then invited for a video call to meet and discuss their potential involvement. Following these interviews, six co‐researchers were selected to be involved in the project. The criteria for the recruitment of co‐researchers included being 18 years old or above; living in a European country; having experience with cancer as a patient or an informal caregiver; having a good command of English, to be able to communicate with the researchers; having the ability, equipment, and willingness to participate in digital meetings; and having the ability and willingness to travel to in‐person meetings. Before launching the project, it was estimated that six co‐researchers would be required, based on the project timeline and estimated workload of the activities. Costs, however, were also a consideration. This number was open to change according to the participation and experiences of the co‐researchers throughout the project [20]. Therefore, after the dropout of one co‐researcher shortly after recruitment, it was decided in agreement with the co‐researchers to continue with five co‐researchers. The co‐researcher group consists of both people with (past experience of) cancer and informal caregivers, and they originate from different countries in Europe. An overview of the co‐researchers' background characteristics is provided in Table 1.

**Table 1:** Background characteristics of co‐researchers.

**Co‐researchers**	**Age range**	**Gender**	**Country of residence**	**Cancer type**
Person with (past experience of) cancer	70–80	Male	Finland	Haematological cancer
Person with (past experience of) cancer	50–60	Female	Italy	Breast cancer
Person with (past experience of) cancer	40–50	Female	Portugal	Breast cancer
Person with (past experience of) cancer	40–50	Female	The Netherlands	Cervical cancer
Informal caregiver	50–60	Female	Ireland	Prostate cancer

Recruitment was finalised in May 2023, and training on the process and content of the research project then followed. The training programme consisted of three online sessions of 1.5 h each: an initial meeting, a second session where the project was discussed in‐depth and a third session reserved for the specific training wishes of the co‐researchers. These trainings were co‐developed with researchers and co‐researchers, based on potential co‐researcher tasks within the project, and specified with regard to co‐researchers' needs [20].

After training, the co‐researchers were connected to several of the project's tasks and activities, based on their skills and preferences. The specific individual roles, tasks and responsibilities of co‐researchers for their activities are defined together with the researchers, evaluated continuously and adapted when necessary, by using the Involvement Matrix [18]. This tool can be used by researchers and co‐researchers to engage in regular dialogue about their ideas, needs and expectations during different phases and activities of the project. Co‐researchers collaborate with researchers through online and in‐person meetings and through email consultations. Support is provided to co‐researchers through the organisation of bimonthly meetings by the researchers who are responsible for PPI in the project. For a more complete and detailed description of PPI activities within the EUonQoL project, and its methodological underpinnings, please consult Engelaar et al. [20].

In this article, we focus on the involvement of co‐researchers in a specific project activity: the development process of the EUonQoL‐Kit. The development process aimed to involve co‐researchers in the discussion of the data that was collected about cancer and relevant domains of quality of life, as well as to collect their reflections and advice on the development of the EUonQoL‐Kit. In addition to their involvement in the actual development process, co‐researchers were involved in the data analysis, the writing of the project report about the development process and the writing of this article as co‐authors.

Besides patients and caregivers, other external actors are involved in the project as members of the Stakeholder Board. The Stakeholder Board was constituted based on a stakeholder mapping exercise. As an initial step, relevant stakeholders working on topics related to the quality of life and mental health of people with cancer, as well as data infrastructure systems, were identified. After the mapping was completed, the identified stakeholders were contacted and invited to join the EUonQoL Stakeholder Board. Currently, the Stakeholder Board is constituted of 11 experts from different geographic backgrounds and with a wide range of expertise. An overview of the Stakeholder Board members is provided in Table 2.

**Table 2:** Overview of stakeholder board members.

**Stakeholder group**	**Country of residence**
Policymaker	Finland
Researcher	France
Researcher	The United Kingdom
Medical society representative	Finland
Medical society representative	Italy
Health economics representative	Germany
Patient organisation representative	France
Social work representative	Greece
Health management representative	Belgium
EU‐funded project representative	Romania
EU‐funded project representative	Portugal

### 2.3 Development Process Description

**Figure 1:**
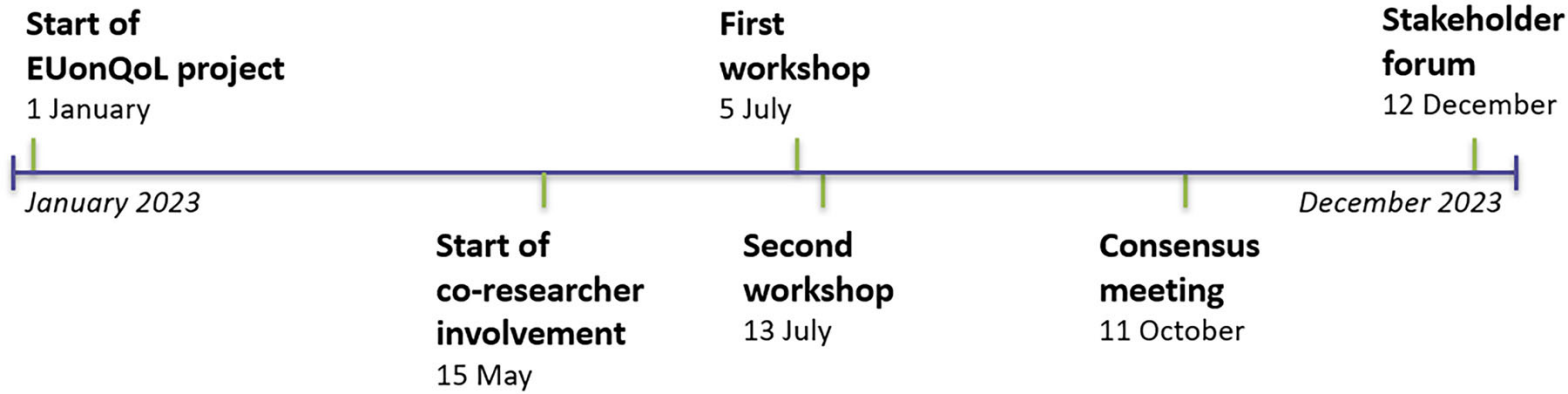
Timeline of the major events during the development process.

The development process of the EUonQoL‐Kit took place between July 2023 and December 2023. It consisted of four major events: two workshops (July), a consensus meeting (October) and a stakeholder forum (December). Figure 1 provides an overview of each event of importance in the development process.

The first workshop aimed to present and integrate the results from systematic reviews, a Delphi study and interviews as input for the draft EUonQoL‐Kit [21, 22]. It was an all‐day meeting with both in‐person and online participation from researchers and co‐researchers. One co‐researcher was present in person, and three co‐researchers were present online. Participants who were present in person were all seated in a U‐shaped set‐up. The workshop was chaired by one of the EUonQoL researchers. The morning began with a brief introduction, followed by an hour and a half of researchers presenting preliminary findings and a session of an hour and a half to bring together the results into the draft EUonQoL‐Kit. The morning session offered limited opportunities for co‐researchers to provide input as it focused mainly on researchers' presentations and discussions. The afternoon session engaged online participants and was more targeted at co‐researchers, starting with a 45‐min recap of the topics that were addressed in the morning session. This was followed by 15 min of discussion. Finally, the potential missing items for the EUonQoL‐Kit were discussed, followed by 10 min of discussion. No specific approach to PPI was applied during this session or the discussions.

The second workshop lasted two and a half hours and aimed to update researchers and co‐researchers on interview and Delphi study results and plan the next steps in the development process of the EUonQoL‐Kit. This workshop was conducted online. Three co‐researchers were present during this meeting. The session began with an introduction to the meeting's aims and structure, followed by an hour and a half of researchers presenting preliminary findings. Each topic consisted of 15 min presentation and 5 min of discussion. In the end, the option to include additional items for patients was presented and discussed, followed by a final 10 min of discussion. No specific approach to PPI was applied during this session or the discussions.

The consensus meeting aimed to collect input from co‐researchers and Stakeholder Board members on which subdomains should be included in the EUonQoL‐Kit. It was an all‐day meeting, allowing both in‐person and online participation. Four co‐researchers were present in person. Co‐researchers and Stakeholder Board members were central to the meeting, as they discussed and rated the potential domains and subdomains for the EUonQoL‐Kit. They were positioned centrally in a U‐shaped set‐up, facing each other. Researchers were seated at the back of the room and listened, took notes and provided clarifications when needed. An independent moderator chaired the meeting, opening the discussion, prompting questions and ensuring everyone had a chance to contribute. The morning began with a welcome, meeting rules and participant introductions. Researchers then presented evidence for including or excluding subdomains for the EUonQoL‐Kit from systematic reviews, interviews and the Delphi study. Following each presentation, co‐researchers and Stakeholder Board members had 35 min to discuss and vote on the relevance and actionability of each domain and its subdomains. The afternoon session featured a short presentation of the voting results and 35 min of reflection and discussion among co‐researchers and Stakeholder Board members. Participants were also invited to suggest new topics and items for the questionnaires. In addition to the official programme, there was also a social aspect to the meeting. A social activity was organised the evening before the consensus meeting, and during the meeting, informal coffee and lunch breaks were facilitated.

The stakeholder forum aimed to introduce the EUonQoL project to a wider stakeholder audience, sense‐check a preliminary version of the EUonQoL‐Kit and discuss the sustainability of the project in the long term with a policy angle. It was a 2‐h online meeting, to which co‐researchers, Stakeholder Board members and a wider stakeholder audience were invited. Four co‐researchers were present during this meeting. The programme started with a word of welcome, followed by presentations about the overall EUonQoL project and the methodological development of the EUonQoL‐Kit. Then, participants were invited to choose between four breakout rooms, each focusing on a specific domain of the EUonQoL‐Kit. In each breakout room, a researcher presented the findings of the research efforts relevant to that domain, that is, systematic reviews, interviews and the Delphi study, enriched by the results of the usability testing which was carried out in the meantime. Participants were encouraged to reflect on these findings during 30 min of discussion that was moderated by another researcher. After the discussions, all participants returned to the main room, where the moderators summarised the major points that had arisen from the discussion in each breakout room.

### 2.4 Data Collection and Analysis

**Figure 2:**
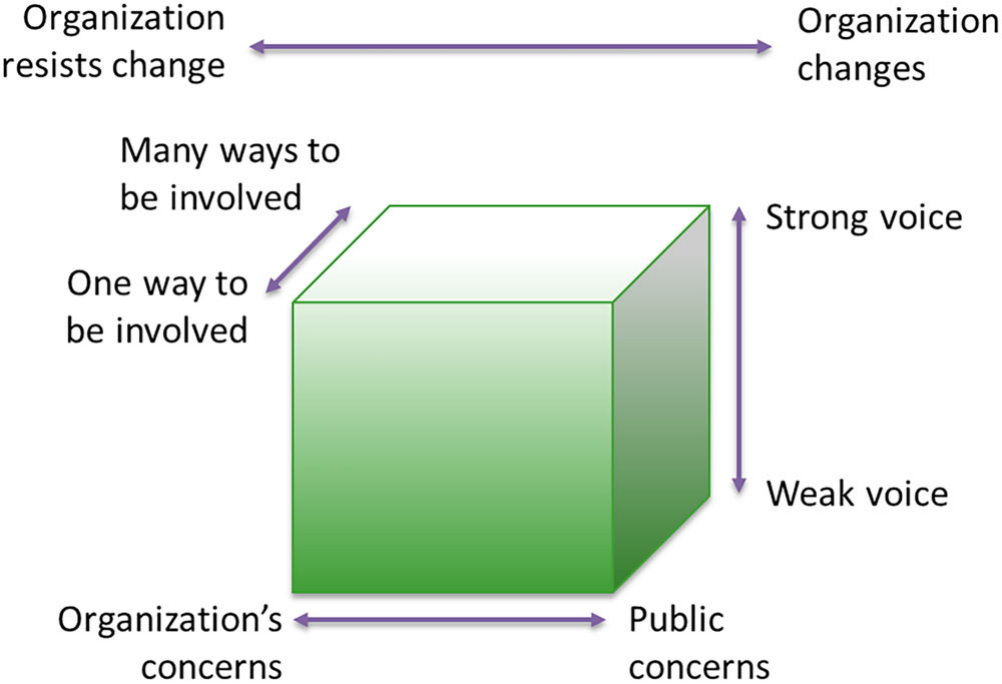
The Cube Framework, adapted from Gibson et al. [24].

We conducted a qualitative analysis of the event documents, that is, meeting agendas, presentation slides, minutes of the events written by researchers and event facilitators, and minutes of regular meetings with co‐researchers before and after the events. We used the Cube Framework by Gibson et al. [23, 24] as a framework to analyse and describe the PPI activities. The Cube Framework describes four dimensions that can be used to characterise and map the dynamics within PPI processes. These four dimensions are depicted as a cube (Figure 2) and include (1) *weak voice—strong voice*, referring to how much influence patients and stakeholders had on decision‐making; (2) *one way to be involved—many ways to be involved*, referring to the number of ways patients and stakeholders were involved; (3) *organisation's concerns—public concerns*, referring to the balance between research and public priorities and (4) the overall dimension *organisation resists change—organisation changes*, referring to the extent researchers were willing and able to make changes to the research based on the input of patients and stakeholders [24, 25]. The document analysis was conducted using MAXQDA software for qualitative analyses. A combination of deductive and inductive analysis was used, in which we used the dimensions of the Cube Framework as a coding framework and added new themes that emerged during the analysis. Two themes were identified during the document analysis that are not addressed in the Cube Framework: the emotions resulting from the development process and new actions resulting from the development process. In this article, the results of these themes are incorporated in the results of the Cube Framework dimensions, due to their close integration.

## 3 Results

### 3.1 First and Second Workshop

#### 3.1.1 Organisation's Concerns—Public Concerns

During the workshops, discussions mostly focused on the researchers' concerns, namely, the technical development of the EUonQoL‐Kit. Co‐researchers sought explanations from researchers on the methodologies used for EUonQoL‐Kit development and domain definitions and provided input on the selection and phrasing of questions within each domain. Co‐researchers also argued why certain topics were important from their perspectives and should be included in the questionnaire, which illustrates the co‐researchers' concerns. Examples of their input included topics such as communication with healthcare professionals, physical fitness and sports, and body image.

#### 3.1.2 One Way to Be Involved—Many Ways to Be Involved

The involvement of co‐researchers was characterised by the fact that the researchers considered them as equal collaborators and, therefore, treated them in a comparable way to researchers. However, the co‐researchers were new to the project and had limited experience in health research, making it challenging to keep up with the information and discussions. One co‐researcher expressed disappointment before the first workshop because information about the meeting had not been shared in time. Due to the strict timeline of the project, it is worth noting that the delay in providing information concerned all participants in the workshop. The time allocated for presenting results to co‐researchers and engaging them in discussions during the meeting was also insufficient for them to fully understand the content and to properly participate in the discussions. Researchers, accustomed to actively participating in meetings, expected the same from co‐researchers, which felt intimidating to the latter and hindered their early involvement. Although researchers asked if co‐researchers understood the information, they did not specifically engage them in discussions, leading to dialogues primarily among researchers. After the workshops, the co‐researchers shared that they found it interesting to participate.

#### 3.1.3 Organisation Resists Change—Organisation Changes

Researchers responded to the co‐researchers' inputs in several ways. They explained definitions of terminology, the methods of developing the EUonQoL‐Kit and reasons for in‐ or excluding certain items from the questionnaire. Additionally, they complimented the co‐researchers on giving good and useful input and wrote down input to give it some more thought later. Follow‐up questions based on the input of co‐researchers were not frequently asked.

#### 3.1.4 Weak Voice—Strong Voice

Co‐researchers mostly gave input when prompted by the researchers but also asked questions and gave their opinions proactively. Co‐researchers reported feeling uncertain, especially during the morning session of the first workshop; they declared that they did not know enough about the project and, therefore, were afraid that they would be asking the wrong questions.

### 3.2 Consensus Meeting

Following the workshops, the researchers responsible for PPI in the project signalled the need to educate the researchers more about collaborating with co‐researchers. For most researchers this was the first time they collaborated with co‐researchers, and it was observed that no specific PPI methods were applied during the meetings to facilitate true involvement. The inexperience of the researchers noticeably hindered the participation of the co‐researchers. Therefore, a training workshop was provided to all the researchers on how to collaborate with co‐researchers, in preparation of the consensus meeting.

#### 3.2.1 Organisation's Concerns—Public Concerns

During the consensus meeting, the emphasis was more on co‐researchers' concerns to tailor the EUonQoL‐Kit to patients' needs and preferences. There were suggestions on refining the wording of subdomains, adding an open question and allowing patients to rate the importance of different items. Most of the co‐researchers' input stemmed from individual experiences, influencing their views on the significance of various subdomains. Key topics addressed included stigma and competition in the workplace, flexibility in return‐to‐work needs and the role of informal caregivers.

#### 3.2.2 One Way to Be Involved—Many Ways to Be Involved

This meeting featured better preparation and involvement of co‐researchers. They had more time to prepare and receive materials in advance, while researchers underwent the training workshop on co‐researcher involvement. The co‐researchers expressed excitement before the consensus meeting, as they were eager to meet each other and the researchers in person. They felt like this was a big step that could potentially change the collaboration in the project in a positive way. More time was allocated for discussions, and co‐researchers were actively involved in the voting rounds. Participants got immediate feedback on the voting results and the input they provided. This session also allowed co‐researchers to propose topics for the EUonQoL‐Kit, giving them the opportunity to offer insights freely. Afterwards, the co‐researchers reported they had enjoyed meeting each other and the researchers. Co‐researchers felt privileged to be seated in the front of the meeting, and the discussion mostly took place among them. Besides the official programme, the social programme was also well‐appreciated. Both researchers and co‐researchers reported that the social activity, coffee breaks and lunch moments were positive experiences that allowed them to talk more with each other about both EUonQoL‐related topics and more personal, informal topics.

#### 3.2.3 Organisation Resists Change—Organisation Changes

The discussion evolved around the co‐researchers, so there was less emphasis on the interaction with the researchers in this meeting. However, in the cases where researchers did get involved in the discussion, it could be seen that they took the training workshop on co‐researcher involvement into account and that their responses to input provided by the participants were different compared to the workshop meetings. In addition to giving explanations about terminologies and methods, researchers responded more openly, for example, by indicating that they should take follow‐up actions and include co‐researchers in this. On occasion, a more open, equal discussion was going on, and researchers asked the participants follow‐up questions.

#### 3.2.4 Weak Voice—Strong Voice

Co‐researchers provided a lot of input. The discussion, which was guided by a moderator, took place among co‐researchers and Stakeholder Board members, resulting in an open, equal dialogue. This made it noticeably easier for co‐researchers to provide input here and make their voice heard than during the workshops.

### 3.3 Stakeholder Forum

#### 3.3.1 Organisation's Concerns—Public Concerns

The stakeholder forum revisited the EUonQoL‐Kit's domains and subdomains, focusing on wider stakeholders' concerns, with discussions divided between technical aspects and content‐related opinions. The diverse audience led to a broader range of topics. On the technical side, participants emphasised the need for clear distinctions between different patient groups (active treatment, survivor and palliative care) and the importance of making the EUonQoL‐Kit user‐friendly and easy to navigate, including an option for free‐text input. Significant content topics discussed included financial toxicity, sex life and intimacy, and communication between healthcare professionals and patients.

#### 3.3.2 One Way to Be Involved—Many Ways to Be Involved

This meeting adopted a similar involvement method to the consensus meeting but was conducted online and included a broader audience. Co‐researchers and stakeholders participated equally in an open dialogue. Co‐researchers were invited to introduce themselves to the audience, either spontaneously or through a prepared quote, which several co‐researchers did. Afterwards, all participants were enthusiastic about the stakeholder forum. Several stakeholders sent emails to the organisation expressing their positive feelings about the meeting.

#### 3.3.3 Organisation Resists Change—Organisation Changes

The discussion points resulting from the breakout rooms were collected and presented to the wider audience, including project researchers. Researchers received the feedback and took this with them in the finalisation of the EUonQoL‐Kit.

#### 3.3.4 Weak Voice—Strong Voice

Much input was provided by the participants, mostly by the wider stakeholders who were new to the project. Co‐researchers provided input to a lesser extent; however, this may be explained by their extensive involvement in the development process to this point and the repetitive element of the different meetings.

### 3.4 Overall Evaluation of PPI

**Figure 3:**
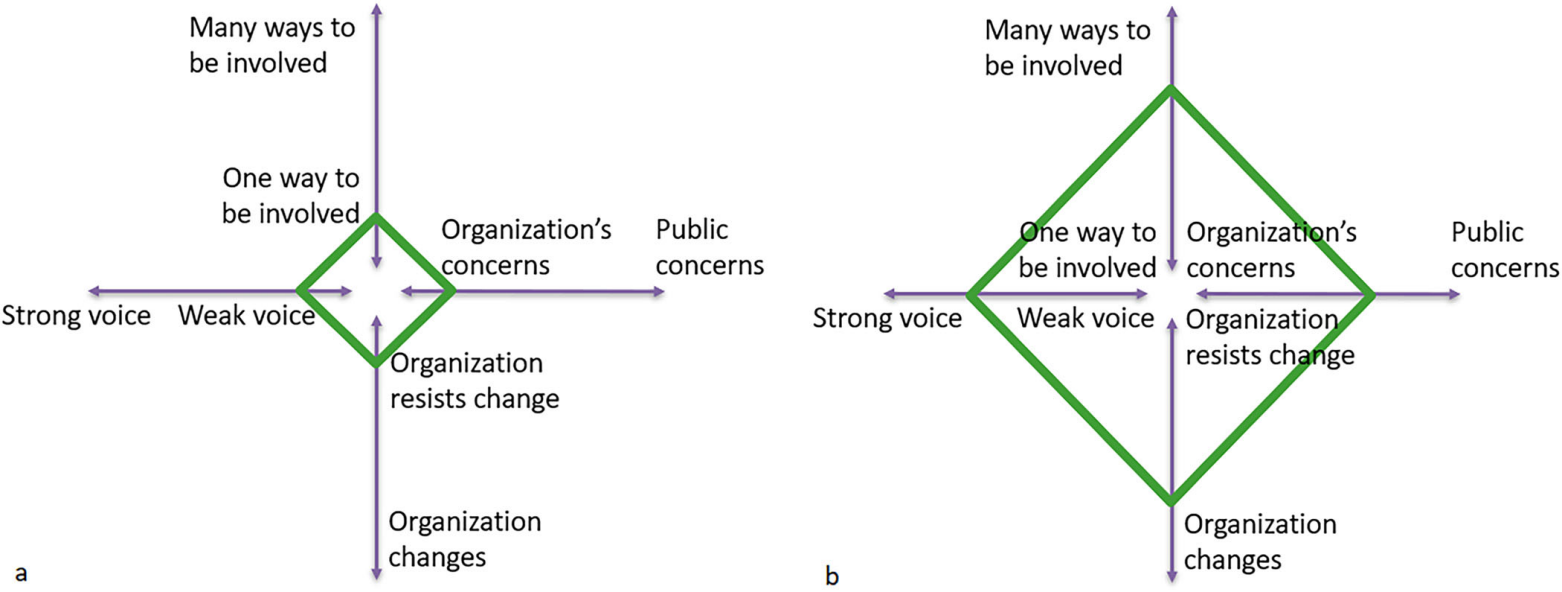
Visual representation of the development in co‐researchers' involvement according to the four dimensions of the Cube Framework, from the initial meetings (a) to the end (b) of the development process.

Across all four dimensions of the Cube Framework, we observed a significant development in the involvement of co‐researchers during the development process. Firstly, within the dimension ‘Organisation's concerns—Public concerns’, discussions evolved from focusing on the researchers' concerns, namely the technical aspects of the EUonQoL‐Kit and its development methods, to emphasising co‐researchers' opinions and experiences to identify key topics for the questionnaires. Secondly, in the dimension ‘One way to be involved—Many ways to be involved’, the methods of involvement were increasingly accommodated to co‐researchers' needs and preferences. In general, during the development process, there was one specific way to be involved: participating in meetings and discussions and providing input during these meetings and discussions. However, we did notice a development in how well this involvement went, and how much the meetings were tailored to the co‐researchers' needs. Initially, researchers treated co‐researchers as equal collaborators, which led to difficulties in their involvement. Researchers' inexperience with PPI prompted the organisation of a training workshop, while co‐researchers' uncertainty led to better preparation and revised planning for future events. This resulted in more opportunities for co‐researchers to express their views and a mutual understanding between researchers and co‐researchers. Thirdly, within the ‘Organisation changes—Organisation resists change’ dimension, researchers became more open to co‐researchers' inputs. They began asking follow‐up questions and giving feedback on how co‐researchers' contributions would be integrated into the EUonQoL‐Kit's development. Finally, all these developments contributed to a stronger voice for co‐researchers in the ‘Weak voice*—*Strong voice’ dimension. There was a shift from co‐researchers providing input only when prompted to actively participate in meetings. A visual representation of these developments is provided in Figure 3.

Co‐researchers were asked to reflect upon the involvement process as well, and they all concurred that the description in this article fits accurately with their experience. Initially, co‐researchers felt somewhat excluded from the discussions, owing to their own uncertainties, a lack of information and the fact that the researchers had already been working on the project for some time. This made them feel ‘invisible’ and not recognised in their role. However, by the consensus meeting, their roles had become more prominent, and the collaboration improved substantially over a few months, thanks to the mediation of the researchers responsible for PPI. These researchers helped co‐researchers gain confidence and guided other researchers in involving them more actively. Regular support meetings clarified project details and fostered team cohesion, enabling co‐researchers to overcome initial obstacles and grow as active contributors. While not having been involved from the beginning surely put them in a weaker position, the co‐researchers now feel they are active contributors to this project. Effective communication and sharing of information have amplified their voices and helped researchers appreciate the patient's perspective. This project demonstrates the importance of planning and integrating PPI from the start, as well as continuously evaluating and adapting it.

## 4 Discussion

The aim of this article was to provide insight into the results of our process evaluation of the involvement of co‐researchers in the development process of the EUonQoL‐Kit. We evaluated the EUonQoL‐Kit development process by analysing how co‐researchers were involved in this, using the four dimensions of the Cube Framework. Additionally, we provide lessons learned for future research efforts beyond the EUonQoL project.

Characterising the involvement of co‐researchers in the development process of the EUonQoL‐Kit along the four dimensions of the Cube Framework provides valuable lessons learned for PPI in general. In line with previous research, we learned that PPI requires significant time, effort and resources to develop effectively within a project [12]. PPI is inevitably a somewhat messy and complex process that requires a lot of work from researchers, patients and the public, since different people are brought together who use different languages, have different experiences and hold different degrees of decision‐making power [10, 26]. At the start of the development process, co‐researchers had only been involved in the project for 2 months and researchers were inexperienced in doing PPI; therefore, we observed that participants had varied understandings of PPI. Based on this observation, a training workshop was provided to the researchers, to teach them how to collaborate with co‐researchers. Additionally, through an iterative process of listening, observing and experiencing, they learned together over time. In line with Dedding [10], we learned that the difficulties we experienced in this process were important aspects of the learning process.

Another lesson learned from our evaluation is the critical difference between equal and equitable collaboration. Treating co‐researchers exactly like researchers*—*as was initially done in the workshops*—*can be counterproductive. Absolute openness and equality may overwhelm co‐researchers with excessive information, leave them confused about their role and burden them with discussions on irrelevant topics [27]. Co‐researchers often lack familiarity with formal research structures, procedures and terminology and contribute from a vulnerable position, frequently as a minority who shares personal experiences. Therefore, their collaboration needs are different from those of researchers. We observed that equitable collaboration can be achieved by actively involving co‐researchers, asking them specific questions and providing space for their input. This includes thoughtful seating arrangements and organising meetings to facilitate their participation. Meeting objectives, agendas, practical details and preparatory materials should be shared in advance to help co‐researchers prepare [27]. Additionally, in line with previous research, we found that support for co‐researchers is vital, such as having a designated, trusted contact person for concerns they might not feel comfortable raising with the wider team [27]. This point of contact monitors the collaboration, addresses difficulties and helps bridge power differences between researchers and co‐researchers. This approach fosters a more inclusive and productive collaboration environment.

PPI is a profoundly relational and interactional process [28], and as a result, we learned not to underestimate the added value of informal contact. The in‐person consensus meeting was a turning point for the relationship between researchers and co‐researchers, as they could get to know each other in the informal setting of a social dinner, a lunch break and coffee breaks. Until then, they had only seen each other in online meetings. The personal connection that was established was found to be important in creating good conditions for collaboration, as the involvement noticeably progressed after having met in person. Without strong connections and a firm grounding, it is difficult to build actual participation practices [10]. Informal moments such as social activities, but also coffee breaks and lunch breaks, help to break down social barriers and increase approachability [27], which is essential to feeling comfortable working together and providing critical feedback. Specifically regarding the vulnerable position that co‐researchers find themselves in and the individual experiences they share, but also to promote collaborative discussion between researchers and co‐researchers.

Finally, we learned that collaborating with co‐researchers in an international, large‐scale context, such as the EUonQoL project, which is a new development, is different from collaborating in national, smaller‐scale contexts. For instance, language and cultural differences play a role. Also, events in the development process mainly took place online. Facilitating online PPI can be an asset, because it supports participation in meetings of introvert patients and stakeholders [13], or those who were previously excluded on the basis of travelling or accessibility difficulties [29]. Additionally, it enables people who live far away from each other to work together, and it accommodates those who are at risk due to clinical vulnerability [30]. However, online PPI can also cause difficulties with regard to digital exclusion if access to technology is limited or if people lack digital skills and with regard to the formal nature of the meetings [29]. We observed that ‘distance creates distance’, meaning that the actual measurable distance between participants makes it harder to feel connected to each other and to the project. One reason for this is the lack of informal contact and small talk during online meetings. This informal connection is necessary to create a good relationship and the right conditions for collaboration.

To support researchers in implementing PPI in similar projects, we have formulated several actionable recommendations based on the lessons we learned (Table 3). These recommendations are useful for other types of research as well, as they cover primary principles of doing PPI, namely regarding the preconditions to be met and regarding the relationship between researchers and co‐researchers. For example, after the COVID‐19 pandemic, there has been increasing online collaboration; however, we have shown that meeting each other in person and informally remains fundamental.

**Table 3:** Recommendations for researchers applying PPI in their projects.

Make sure that everyone is well‐prepared regarding the content of the research, as well as the method of applying PPI. Provide training to both researchers and co‐researchers.
Appoint researchers who are specifically responsible for the PPI activities and have them support the co‐researchers through meetings and email contact. Also, make sure those researchers are easily accessible and approachable to the co‐researchers.
Schedule plenty of time and space in the process to develop the collaboration further. Avoid tight timelines.
Strive for an equitable collaboration, by finding out what co‐researchers need in the collaboration and providing this to them.
Schedule moments of in‐person, informal contact early on in the process and during the entire project. Make time for informal contact in online meetings as well.

This study has some strengths and limitations. Firstly, to our knowledge, this is one of the first projects where PPI is applied on such a large, pan‐European scale. We have provided an extensive and critical process evaluation of the involvement of co‐researchers in the development process of the EUonQoL‐Kit, with considerable attention to the co‐researchers' perspective. The challenges we encountered and described can help other researchers to overcome the same challenges. One of the limitations of the study is the relatively small number of co‐researchers involved in the project. However, PPI is not necessarily about representativeness but about adding new, equally important perspectives to research and decision‐making. Regarding representativeness, a Delphi study and patient interviews were conducted as input for the EUonQoL‐Kit, and the pilot study will enrol a large number of patients throughout Europe. Lastly, this article covers a process evaluation and, therefore, there is a lack of specific examples of the contributions that co‐researchers made to the EUonQoL‐Kit. However, these findings will be included in future publications.

## 5 Conclusion

In conclusion, PPI in the development process of the EUonQoL‐Kit was a learning process. Where initially researchers and co‐researchers felt some discomfort in working together and were exploring the best way to do so, later there was a mutual connection and understanding established that benefitted the collaboration. Factors that helped with this were good preparation of all parties, provision of support by trusted researchers who were specifically responsible for the PPI activities, taking the time and providing the space in the process to develop the collaboration further, striving for an equitable collaboration, and realising the importance of informal contact. We will take these lessons learned in the next stages of the EUonQoL project, which will involve a pilot study to validate the EUonQoL‐Kit and dissemination of the study results. By doing so, we strive to at least maintain the same level of involvement and collaboration that we have now achieved and to work even more towards a collaboration that is desirable and pleasant for everyone involved and that leads to jointly supported outcomes. Additionally, with this article, we strive to support and inspire researchers engaging in PPI in quality of life research, oncological research and health research in general. Future PPI efforts should benefit from the lessons and recommendations we have described here, by incorporating these principles from the start to facilitate successful collaboration between researchers and co‐researchers.

## Author Contributions

Merel Engelaar was responsible for the conceptualisation of the article, the data analysis and the writing of the original draft, as well as incorporating input from the other authors. Femke van Schelven, Carina Dantas, Caitriona Higgins, Tapani Kalmi, Inke Minnée‐van Braak and Laura Pinnavaia were involved in the conceptualisation and data analysis as well and provided feedback on draft versions of the article. Nanne Bos, Marion L'Hote, Clémentine Rialland, Norbert Couespel and Jany Rademakers were involved in the conceptualisation of the article and provided feedback on draft versions. Giovanni Apolone, Cinzia Brunelli, Augusto Caraceni, Montse Ferrer, Mogens Groenvold, Stein Kaasa, Gennaro Ciliberto, Claudio Lombardo, Ricardo Pietrobon, Gabriella Pravettoni, Aude Sirven, Hugo Vachon and Galina Velikova provided substantial contributions to the conception and design of the EUonQoL project, including the PPI approach described in this article, and provided feedback on the final draft. All authors read and approved the final manuscript.

## Disclosure

The views and opinions expressed are, however, those of the authors only and do not necessarily reflect those of the European Union or the European Health and Digital Executive Agency (HADEA). Neither the European Union nor the granting authority can be held responsible for them.

## Ethics Statement

The co‐researchers all signed a project agreement that includes the following articles: services to be provided, confidentiality, period of agreement and termination, fee and payment schedule, liability and final provisions. This is an extended form of informed consent.

## Conflicts of Interest

The authors declare no conflicts of interest.

## Data Availability

The data that support the findings of this study are available from the corresponding author upon reasonable request. Data will be anonymised before being shared.
